# Coping with digital market re-organization: How the hotel industry strategically responds to digital platform power

**DOI:** 10.1177/10245294211055612

**Published:** 2022-01-13

**Authors:** Philip Balsiger, Thomas Jammet, Nicola Cianferoni, Muriel Surdez

**Affiliations:** 27214 University of Neuchâtel, Switzerland; School of Social Work Fribourg, 000 University of Applied Sciences and Arts Western Switzerland (HES-SO), and University of Neuchâtel, Switzerland.; 000 State Secretariat for Economic Affairs (Seco) and University of Geneva; 27211 University of Fribourg, Switzerland

**Keywords:** Platform capitalism, market, strategic response, hotel industry

## Abstract

How do organizations in a sector where powerful platforms have emerged cope with the new constraints and opportunities that platforms induce? A growing number of studies highlight the power of digital platforms to re-organize markets and thereby create new forms of dependence. But there are also indications that organizations are capable of countering platform power especially by demanding their regulation. This paper expands this view to investigate also strategies at the organizational level. It draws on the algorithmic game studies of strategic responses to environmental changes to study how organizations strategically respond to the rise of digital platforms. To show organizations’ capacities to cope with the new digital market environment, we use a qualitative case study of the Swiss hotel sector and its reactions to so-called online travel agencies, based on interviews with hotel managers and professional representatives. We distinguish between three types of hotels—small family-run, luxury, and chain hotels, and identify three types of strategic responses: bypassing, optimizing, and mitigating. Contrary to a platform power perspective, we find some evidence for organizations’ capacity to keep platforms at bay, by limiting dependence through mitigation, and platforms’ reach through bypassing. Hotels also learn to “play the algorithmic game” and take advantage of platforms’ technological affordances, but such strategies seem to accommodate platform power rather than countering it. Finally, we find that hotels with fewer resources (small family-run hotels) are less equipped to counter platform power, suggesting that platforms risk fostering existing hierarchies and segmentation in markets.

## Introduction

Over the past decade, digital platforms have emerged in many sectors and tend to disrupt existing markets. As a new form of economic organization (Kenney and Zysman, 2016), platforms create new market hierarchies and control market transactions. Platforms re-organize markets (Ahrne et al., 2015) to their own benefit by skimming a rent on transactions (Kirchner and Beyer, 2016). Many observers proclaim the coming of a new era of “platform capitalism” (Srnicek, 2017) characterized by a few dominant platforms that algorithmically govern markets and dictate market conditions on all market participants, be they workers or organizations. This perspective of platform power—what Vallas and Schor (2020) call the “digital cage view”—is pervasive in both academic and popular accounts, pointing at new forms of algorithmic control and data-driven surveillance capitalism (Zuboff, 2019).

However, this “platform power” picture runs the risk of underestimating the diversity of platforms, their unequal effects on different market participants, and the capacities of the latter to develop strategic responses. There are indications that platforms are not as powerful as to single-handedly drive organizational change without being contested. Collective struggles over regulation of many platforms are taking place in many countries (Kirchner and Schüssler, 2020) and may limit platforms’ capacity to organize markets. Some recent studies (Christin, 2017; Velthuis and Van Doorn, 2020) also indicate the potential of market participants to develop strategies that allow them to individually adapt to the new environment of platforms and reduce their dependence on them, limiting the reach and forms of platforms’ re-organizing of markets.

So far, this question has been addressed in the realm of gig workers (Vallas and Schor, 2020: 16.6–16.7; Velthuis and Van Doorn, 2020) but much less for cases where organizations get “platformized”—existing production or service markets where one observes the rise of new digital intermediaries. This paper empirically investigates such a configuration, focusing on organizations’ capacities to develop strategic responses to environmental changes (Espeland and Sauder, 2007; Oliver, 1991; Pollock et al., 2018; Velthuis and Van Doorn, 2020). Do platforms similarly affect all types of organizations? What kind of responses do organizations develop, and how do these responses limit platforms’ capacities to re-organize markets?

To answer these questions, we propose a qualitative study of the hotel industry in Switzerland. The hotel sector, which constitutes a strategic economic sector in Switzerland, is an ideal case study since this industry was “disrupted” early on by a specific kind of platforms called Online Travel Agencies (OTA) like Booking.com or Expedia (Cetin et al., 2016b; Lam and Law, 2019; Melis and Piga, 2017). Functioning as new intermediaries (Bessy and Chauvin, 2013), these platforms have supplanted traditional booking methods for a large part of customers. Hoteliers are thus facing new challenges and management practices. Based on interviews with hoteliers, the paper first describes the differentiated effects of platforms on hotel types before analyzing the industry’s strategic responses to the rise of platforms. We find that in parallel to efforts of regulation taken by the professional associations, which concerned only the specific issue of price competition and had only limited effects in the Swiss case, hotels developed strategic responses at the level of individual organizations to cope with this absence of regulation. We develop a typology of the range of the responses they use: bypassing, optimizing, and mitigating.

The study contributes from a perspective of economic sociology to the rising literature on the digital economy, and more particularly on the power of platforms to impose a new digital market order (Kirchner and Beyer, 2016) by re-organizing markets (Ahrne et al., 2015). We expand on existing studies whose main focus is on digital workers by looking at a sector where *organizations* (i.e., hotels) are subject to the rise of platforms as market intermediaries. Through this lens, we will nuance the “platform power” perspective, finding some evidence for organizations’ capacity to keep platforms at bay. We find in particular that hotels are able to limit the reach of platforms by developing strategies of bypassing and to limit their dependence by selling on different platforms at the same time. We also show that they learn to “play the algorithmic game” and can leverage the technological affordances of platforms, but such strategies seem to accommodate platforms more than countering them. Finally, we see strong differences of platform effects depending on hotel types; the most powerful of them—chains—are sometimes capable of reversing market hierarchies. As a consequence, our study suggests that although market participants deploy strategies to counter platforms’ attempts to re-organize markets, platforms risk fostering existing hierarchies and segmentation in markets.

While the results from a single case study are by definition limited, they allow for a deeper understanding of the effects of platforms in the specific environment in which they are embedded. Furthermore, the types of strategic responses and the underlying mechanisms identified are likely to be important in other sectors, too. Examples may include the restaurant sector where restaurants are facing the expansion of meal delivery platforms, or the legal services sector where firms are competing with platforms offering standardized services and new forms of expertise (Christin, 2017; Flood and Robb, 2019).

The first section further develops the theoretical framework, before explaining case selection and methods. The ensuing empirical analysis is organized as follows: We start with an account of the emergence of platforms in the hotel sector and the different perceptions thereof according to hotel types. We then go on to describe the various strategic responses hotels developed, starting with a short account of collective action before detailing the individual strategies. We end by assessing how organizations of the hotel sector manage to counter platforms and by discussing the contributions of this case study to the literature on the power of platforms to re-organize markets.

## Theoretical framework

### Platformization of markets

Digital platforms have been described as the new organizing principle of capitalism (Kenney and Zysman, 2016; Srnicek, 2017; Staab, 2019). As software-based programs able to create network externalities and to govern an ecosystem of actors (De Reuver et al., 2018; Hein et al., 2020), digital platforms derive their power from their technological and economic features. Many studies have indicated how the rise of digital platforms raises various questions of power, in particular with regard of their socio-political effects in terms of changes to the public sphere and the shaping of democratic debates and civic practices in the “platform society” (Lynskey, 2017; van Dijck et al., 2019b). Using an economic sociology framework, this paper’s focus is more specifically on the issue of platform power in the process of market re-organization by platforms, analyzing the mechanisms of platform power and the strategic responses to it.

Moving away from the idealized market view that all actors benefit from the value creation generated by digital platforms, recent streams of research question who captures this value (Hein et al., 2020: 98). To untangle “the patterns of dependency that tie platforms, end-users, and complementors [actors that propose services or products on the platforms] together” (van Dijck et al., 2019a: 8), sociological approaches look at the level of control platforms exert over the whole ecosystem through their gate-keeper role with regard to market and/or information access (Lynskey, 2017) and describe the (more or less permissive) forms of governance (Vallas and Schor, 2020) they install.

As “digital, databased, and algorithmically structuring socio-technical infrastructures that exchange information, coordinate communication or organize work, offer a wide range of services, or distribute digital and non-digital products” (Dolata, 2019: 183), digital platforms are not just market actors but market *organizers* (Ahrne et al., 2015; Kirchner and Schüssler, 2019). They structure the transactions taking place within the digital environment that they constitute with an economic model based on skimming a rent on market transactions (Kirchner and Schüssler, 2019). As a consequence, they act as typical market “profiteers” (Ahrne et al., 2015). The growing literature on the platform economy mostly paints a picture of a newly emerging hierarchical structure of digital markets creating new forms of dependence with far-reaching effects on local market institutions and producers or service providers (Dolata, 2019; Kenney and Zysman, 2016; Nieborg and Poell, 2018; Staab, 2019). This aspect is especially pronounced since platforms seem to benefit strongly from network effects, which suggests a tendency of monopolization inherent to the platform economy (Srnicek, 2017).

Toward consumers, platforms act as “capturing devices” (Cochoy, 2004; Trompette, 2005). This means that they seek to attract users and maintain them within the digital ecosystem they provide by applying “lock-in strategies” that make it both unnecessary and costly to switch to a different platform (Dolata, 2015). They do so in particular by expanding their portfolio to cover the market as fully as possible, by facilitating market transactions through intuitive and efficient tools, and through individualized algorithmic recommendations enabled by their processing and data management power (Orlikowski and Scott, 2015). Thanks to the technology, differentiated data profiles emerge when users navigate on the platform, and these profiles “contribute to the algorithmic refinement and quality improvement of the platform-specific information, search, evaluation, and interaction systems” (Dolata, 2019: 188).

On the other side, towards producers or service providers, platforms exert control by imposing their conditions on market transactions (Kenney and Zysman, 2016; Staab, 2019). This concerns all aspects of these transactions: standardization of the presentation of the offer, categorization of the goods exchanged, price determination through the setting of commissions, as well as the general “terms and conditions” such as, for instance, return or cancellation policies. If successful in their attempt to re-organize markets, platforms therefore gain a position where they control access to markets, dictate conditions of exchange and shape products, product categories, and evaluation systems, and where both consumers and service providers/producers become increasingly dependent on them (Kirchner and Beyer, 2016).

### Strategic responses to platform power

This “platform power perspective” has been widely adopted, with much of the existing literature describing how platforms create new powerful forms of dependence on markets (Dolata, 2019; Kenney and Zysman, 2016; Kirchner and Beyer, 2016; Srnicek, 2017; Staab, 2019). But with the exception of the growing body of empirical research on the “gig economy” (Anwar and Graham 2020; Raj-Reichert et al., 2020; Ravenelle 2019; Vallas and Schor 2020; Wood et al., 2019), systematic studies of platforms effects on market participants are still scarce, especially for cases where *organizations* are subject to the rise of platforms. Here, the power of platforms and their ability to impose a “platform logic” (Kirchner and Beyer, 2016) on markets often remain a theoretical statement, and more empirical work is needed to show the actual effects of platforms and the reactions of market participants.

There are indications that platforms are not always as powerful as the literature suggests. Market participants may resist the re-organization of markets by platforms. Mostly, this has been documented at the political level, where studies on regulation struggles around some of the most visible platforms like Uber or Airbnb (Collier et al., 2018; Culpepper and Thelen, 2019; Serafin, 2019; Thelen, 2018) reveal that platform power can provoke strong political resistance (Woodcock and Graham, 2020). Attempts to regulate platforms are underway in many sectors and countries (Kirchner and Schüssler, 2020) and are generally pursued collectively by industry organizations as well as by political bodies who nevertheless face difficulties to determine the economic or political components of their legal action due to the multidimensional development of platformed environments (Lynskey, 2017).

Yet, platforms’ capacity to re-organize markets is also challenged at the level of organizational “strategic responses” (Oliver, 1991). Market participants may develop strategies that allow them to adapt to the new environment of platforms and undercut their dependence on them. Studies on the gig economy have shown workers’ agentic capacity to resist platform control and creatively adapt to their logic, for instance, by switching between platforms (Velthuis and Van Doorn, 2020) or playing their algorithms (Vallas and Schor, 2020: 16.6–16.7). Similarly, rather than being passively submitted to institutional pressures, we can expect organizations to be able to strategically adapt to this. Studies on the reactivity of firms to the evaluative infrastructures (Kornberger et al., 2017) of rankings and ratings have shown strategic responses developed by organizations, for instance, by reacting to customer reviews in order to protect their market identity (Beuscart et al., 2016; Wang et al., 2016). In general, however, such strategic reactions tend to reinforce the disciplining effect of market devices (Espeland and Sauder, 2007). Beyond reactions to evaluative devices, some studies specific to the hotel industry also indicate that organizations seek to limit their dependence on platforms by reinforcing alternative distribution channels, as well as by using platforms’ technological affordances in their favor (Alrawadieh et al., 2021; Cetin et al., 2016b; Lam and Law, 2019; Melis and Piga, 2017). However, this literature often focuses on one hotel type and on describing the balance of advantages and disadvantages procured by OTAs rather than examining coping strategies and analyzing the broader question of the re-organization of markets and platform power.

In this paper, we seek to identify the strategies hotels develop to cope with digital market re-organization imposed by platforms. By locking-in consumers into their ecosystem and providing efficient access to them, platforms become able to impose their conditions on market participants (Cutolo and Kenney, 2020); at the same time, firms will seek to reduce platforms’ power over them. We expect that not all market participants will be equally affected by platforms. Studies of the gig economy indicate strong differences between workers with respect to their dependence on platforms and their capacities to adapt to them (Naulin and Jourdain, 2020; Schor et al., 2017; Vallas and Schor, 2020). It is therefore important to study the effects and responses to platforms for different types of organizations. Depending on their position in the market and the resources at their disposal, not all market participants will be equally equipped to develop the skills to successfully navigate the new digital market environment. In sum, while platforms have been shown to have strong disruptive power, we expect them to have unequal effects and market actors to develop strategic responses to counter and/or adapt to the new environment. Reactions are likely to differ depending on the structural position, resources and skills market participants dispose of (Cutolo and Kenney, 2020).

## Case and methods

Our case study is the hotel industry in Switzerland. Not only is tourism a longstanding and important economic sector in Switzerland (Humair and Tissot, 2011), but it is also diversified, with two main types of tourism, leisure and business, and the maintenance in certain regions of small hotel structures run by independent owners. This case is therefore relevant to our research question which aims to capture the plurality of responses to the expansion of platforms.

The hotel sector has been strongly affected by the development of two kinds of digital platforms: OTAs as intermediaries where hotels use platforms to offer their products to clients and the more recent peer-to-peer platform Airbnb (Gazzoli et al., 2008; Kirchner and Beyer, 2016). From an economic sociology perspective, the main feature of OTAs is their capacity to re-organize the traditional accommodation market, whereas Airbnb expands this market by adding new competitors (Kirchner and Schüssler, 2019). Because the two kinds of platforms pose different challenges^1^, this paper focuses exclusively on OTAs to study strategic responses. US and European companies such as Expedia, Hotel Reservation Service(HRS), or Booking.com are typical global intermediary platforms, who let consumers browse hotels by city or region, compare offers, and purchase rooms. By far, the most important platform in the Swiss market today is Booking.com, with a market share among OTAs of around 75%, followed by Expedia (13%) and HRS (5%) (Schegg, 2019). In 2018, OTAs represented 28% of all reservations in Switzerland; for 44% of hotels, more than 30% of their reservations came from OTAs, and for 20% of them even more than half of all reservations (ibid.).

This paper presents results of a broader research project on the effects of digital platforms on professions and markets in the hotel sector in Switzerland, centered on both professional associations’ reactions and hotels’ reactions. Two contrasting regions were selected—an urban center with both business and leisure travel, and a mountain region with mostly leisure travel. We expect that the actors active in the urban aera, characterized by more diversified forms of tourism, better traveling facilities, and a higher presence of hotel chains, will be more inclined to use the opportunities offered by OTAs, while the actors based in the mountain zone with a higher importance of regular patrons are expected to have a more conservative perspective. The qualitative fieldwork collected and analyzed in-depth interviews with hotel owners and/or managers (*n* = 10), representatives of the sectoral and professional associations (*n* = 9, of which 3 are or were themselves hotel owners and/or managers), distribution companies (*n* = 2, one of whom is an OTA), IT services companies (*n* = 4), and field experts (*n* = 4). Interviews were conducted face to face between April 2019 and January 2020 at the interviewee’s workplace and lasted between 1 and 2.5 h.

In order to grasp the range of strategic reactions to platforms at the level of hotels, the results discussed in this paper focus on the interviews conducted with hotel owners and/or managers. For practical reasons, we started our study with the representatives of the professional associations. In order to test if hotels have different strategies according to their features as organizations, we asked these representatives to refer us to 10 hotels corresponding to the classification of three to four stars, which is the most frequent category in both regions, and coping differently with the OTAs. This led to a diversified sample of hotels studied. Based on our analysis of the specific challenges, perceptions, and strategic reactions of hotels, we grouped them into three categories that combine characteristics of the property and customer segment: small, family-run middle-class hotels; chain hotels; and independent luxury hotels. These categories can be considered as characteristic of the hotel sector in Switzerland. In the urban center, three hotels belong to or are franchised by a chain, a category that is uncommon in the mountain region. Accordingly, mountain hotels are far smaller than the urban ones, and five hoteliers in the mountain region are owners and managers of their establishment, while in the urban center, four out of five hoteliers are managers or member of the upper management.

Coding and analysis followed an iterative process of abductive analysis (Tavory and Timmermans, 2014), starting from a theoretically informed reading, which led us to a thematic open coding of the interviews through which the analytical categories emerged (Miles et al., 2014). Coding was done by the two authors who also conducted the interviews (with the help of the software Atlas-ti). For this paper, we focus our analysis on the codes related to the relationship between hotels (and to a lesser degree, their professional associations) and OTAs. To analyze strategies, we at first coded all actions and practices interviewees mentioned with regard to OTAs, as well as the skills, technical tools, and material devices that were associated to them (such as, for instance, IT skills, channel manager (CM) software, and specific labels). In a further analytical step, we grouped these strategies together. First, we distinguished between strategies taking place off or on platforms. Second, for the strategies taking place *on* platforms, we distinguished between the goals of the use of platforms: either maximizing the profit from the platform or minimizing its undesired effects. This led us to our categorization of bypassing, optimization and mitigation and of the subgroups presented in the analysis. Finally, we related both perceptions and strategies to our “hotel type” code, to see to what extent they vary with regard to specific features of hotels and/or geographic region.

### From opportunity to disillusion: Evolving perceptions of OTAs

In the first years of their existence, OTAs were relatively marginal players in the Swiss hotel market. This changed quite quickly towards the end of the 2000s, when within a few years, Swiss hotels put their rooms massively on OTAs. There are indications that this was linked to contextual factors—hoteliers mention the financial crisis of 2008, which had as a consequence, a strengthening of the Swiss franc that provoked a crisis in tourism. In this context the promises of OTAs were appealing: they were a means to promote hotels’ international visibility without initial fixed costs (since the fees are based on commissions collected on revenues generated by bookings) and thus to liberate oneself from dependence on traditional “off line” travel agencies and accelerate cash-flow by appealing to consumers directly. Particularly, small family-run hotels saw great advantages: before, they hardly had opportunities to promote their offer toward non-European tourists and did not have sufficient financial means to invest in huge technical infrastructure or international marketing. At the same time, the OTAs themselves spent much effort in actively recruiting hotels.

However, what at first looked like a way towards emancipation from travel agencies in favor of a more direct appeal to customers worldwide turned out to establish a new form of dependency for the hotels. The consequences of this process of “remediation” (Cetin et al., 2016a) is obvious even for the manager of a big chain hotel of the urban center. To him, “the presence that I have internationally with such a web page, that’s enormous. As an individual hotel, I can never achieve this without Booking.com.” Yet at the same time, this also means that he “can’t live without Booking.com.” Almost all hoteliers shared this view, stating that they could no longer run their business without being connected to OTAs.

This situation of increased platform power creates three main concerns among hoteliers. First, hoteliers fear to lose their control over key features of business policy, with platforms dictating all major terms of *transaction conditions*. Some of these conditions are seen as positive, such as a more rapid cash-flow. But others are perceived as imposing unfavorable conditions and infringing the hoteliers “commercial freedom,” in terms of cancellation policies and of price. The latter is related to the commissions OTAs charge on all transactions^2^, and which increase the ratio of fixed costs to sales and change the cost structures for hotels. Furthermore, through so-called parity clauses, OTAs seek to prohibit hotels from offering lower prices on other distribution channels. Such policies severely limit the ability of hotels to vary their room prices.

Second, hoteliers are concerned about their ability to control *relations with customers* without passing through a platform and its algorithm. They denounce OTAs handling of customer contact details, which are only partially shared with hotels. As a result, hoteliers cannot directly get in touch with guests before they arrive.

A third issue at stake is the way platforms *algorithmically rank* hotels and thus present them in a certain order to potential customers. This algorithmic sorting creates new value hierarchies whose precise mechanisms are unknown to hoteliers (Stark and Pais, 2020). Customer reviews play a role therein, and hoteliers fear the impact of negative reviews on their ranking on the platform. The opacity of the algorithms that govern the establishment of rankings is a recurring reason for complaints among hoteliers who are subject to it (Cardon, 2015; Orlikowski and Scott, 2014). In Switzerland, six hotels out of 10 consider that OTAs do not communicate in a transparent and understandable manner how the rankings are established, nor how they can influence their position in these rankings (Schegg, 2019). Overall, the issues raised by the hoteliers are closely related to what the literature has called the gate-keeper role of the platforms (Lynskey, 2017): the cost of accessing the market (commissions on transactions) and access to information (information on customers and operations of the algorithm).

### Strategic responses to the rise of OTAs

The increasing platform power with its new form of dependency led hoteliers to develop a range of strategic responses at the collective and individual level. At the collective level, it concerned the classic aspect of demanding price regulation from political or regulative authorities. The contested element were the so-called price parity clauses. Through these clauses, which were an integral part of the standard contract, OTAs prohibited the hotels from offering a lower price for the same room on any other distribution channel—including not just the hotel’s own website, but even for a walk-in customer. Price parity clauses are thus a way through which OTAs seek to influence price setting for the market as a whole, by imposing rules whose validity goes beyond their own platform, and by threatening to punish the actors who would deviate from it. If spotted, breaches could lead to sanctions by OTAs—notably an algorithmic downgrading in the ranking based on the performance of the hotel and its evaluation by the customers. Hoteliers considered that these clauses seriously impaired their organizational autonomy. Nearly all hotels and their professional associations agreed to challenge these clauses legally and politically, with an appeal at the Federal competition commission^3^, which was then followed by a parliamentary interpellation that still has to be implemented.

On this point, where there was agreement among hoteliers, the professional associations took political action to counter platform power through state regulation, mobilizing an antitrust argument. But this also means that the professional associations tried to counter platforms only on one quite specific point, building on the most common form of market regulation in terms of price. The collective response does therefore not cover all the issues of concern discussed in the previous section. In all these other aspects, OTAs can keep exerting their power and the capacities of regulation are uncertain, not least because platforms introduce innovations for which there are no clear regulatory frames (Lynskey, 2017). It is in these spaces that the strategic responses at the level of individual organizations are developed. In addition, the legal and political process engaged by the hotel sector’s professional associations also had a disinhibiting effect on the hotel industry, by encouraging hotels to undertake various strategies with regard to OTAs: “The hoteliers have gained confidence and dare much more now to take risks towards Booking.com than they have done in the past. That’s positive.” (Representative of interregional professional association)

The “risks” this representative refers to are individual strategies developed by hoteliers to favor direct bookings. Sometimes, it seems that they simply do not respect the parity clauses, knowing that in most cases, OTAs do no longer enforce their rules actively. But more importantly, they develop offers that respect the rules imposed by the platform but are distinct from what is offered on the platform. Such strategies aim at bypassing platforms. While a minority of hotels decided to avoid platforms completely, we find that most of them combined bypassing with strategies of optimization and/or mitigation. We now turn to analyze this strategic repertoire in more detail.

### Bypassing

Most hotels do not put their whole inventory of rooms on OTAs. Yet even for the rooms that are bookable on OTAs, hotels often would prefer that customers book them directly whether on the hotel’s website or by calling or e-mailing, in order to avoid or at least attenuate their dependence on OTAs. By encouraging direct booking, hotels look for diminishing the amounts of commissions paid to OTAs. They also attempt to regain room for maneuver in setting the conditions of the transaction with the clients. Essential for this dynamic is a user-friendly digital booking interface. Indeed, in particular for small family-run hotels, it is often very difficult to keep up with the platforms in terms of ease of online booking, while luxury and chain hotels have the resources to build or acquire a user-friendly website equipped with a booking engine.

We find a range of bypassing strategies here that all build on the development of distinctive forms of qualification and valuation (Beckert and Aspers, 2011; Beckert and Musselin, 2013) that are different from what one can find on OTAs. By developing these offers, hotels enter into a competitive dynamic induced by platforms. However, not all of these offers are new; moreover, they are not equally distributed among hotels and clients. Depending on the types of hotels, different strategies of qualification and valuation are used: special offers, quality labels, and loyalty programs.

Small hotels often simply develop *special offers* on their website such as packages that include small perks that cannot be booked on OTAs—for instance, rooms that include a welcome drink, free spa use, or special offers for families. Such special offers are a way to avoid the platforms’ algorithmic controlling and sanctioning in case of the price parity clauses’ non respect.

One particular example is provided by a hotel located in the mountain region. The owner explains that he developed fasting packages in order to extend average stay durations:

Since the beginning of this year we also offer a program based on fasting stays in addition to our already existing phytonutrition stays [...] Stays are shorter and shorter now in Switzerland. [...] How can we fight against this phenomenon? Well, we have to create long stays! A fasting stay lasts six days. […] With the product I have, I can go all over the world looking for people who don’t give a damn whether the franc is like this or the euro is like that.

“Value packages” can also include experiences related to the touristic environment, such as, for instance, ski passes. Here, hotels can take advantage of their local networks, which allow them to develop such offers more easily than OTAs.

Another way of creating distinct value is through *quality labels*. Traditionally, hotels are qualified through the star systems, although customer evaluations seem to increasingly challenge this (Cardon, 2015; Orlikowski and Scott, 2014). Yet as in all markets, other forms of quality labels exist in the hotel sector, too, and they have become increasingly present in recent times to distinguish specific market niches. On the one hand, such distinctions are not easily visible on OTAs, where hotels are sorted according to other criteria. On the other hand, these labels, which indicate specific qualities, may attract customers who will then be guided to the hotel’s own website. The Swiss Historic Hotels network, for instance, was founded in 2004 and promises stays where the guest can “time travel” in well-conserved historic hotels certified by experts. One mountain hotelier, owner of a luxury hotel, told us that “a lot of clients come through this way. We tried to integrate a group or a travel guide that presents the hotels in a bit different manner and which characteristics match well with ours.”

Another example is the international *Relais & Châteaux* label. Such a label can substitute the platforms in a marketing perspective by giving international visibility beyond national borders for the segment of the market based on high quality and luxury services, as a hotelier from the mountain region summarizes:

For us it’s very important to really stand out from the competition. [...] *Relais & Châteaux* is one of the few independent labels that really puts the emphasis on quality, on cuisine. It’s a good stamp for asserting the quality of a hotel. [...] We have a lot of clients who book through *Relais & Châteaux*. That’s great and very interesting for us.

Contrary to packages or experiences, these quality labels rest on third party actors, often professional associations gathering hoteliers or other touristic actors, such as tourism offices, transport and cable car enterprises, firms editing guidebooks, and so on. Selective criteria exist, and hotels usually have to pay a fee to be part of such a grouping. However, quality labels are not reserved to luxury hotels—other examples of labels issued by the main professional associations, such as Family Hotels or Bike Hotels, cater to different market segments and niches.

A last strategy of qualification and valuation used for bypassing OTAs are *loyalty programs*. While most hotels will make attempts to win customer loyalty, explicit loyalty programs exist in particular at chain hotels. Chains as such can be seen as type of quality label, and with their specific loyalty program, they combine a label approach with a package approach. An owner says that in his hotel (which is member of an international chain), 30–35% of the guests are beneficiary of a loyalty program:

Loyalty programs have to give a calculable advantage to clients. If as a client, I arrive through Booking.com I have this price. If I arrive through the Club [the hotel’s loyalty program], I have the same price, but I have a bigger room, I have soft benefits like a welcome drink, free drinks, etc.

With loyalty programs, the more often the clients come, the greater the value they get. This play around value instead of price is what characterizes all bypassing strategies.

### Optimization

For all their attempts at bypassing, OTAs remain essential for most hotels. But for the rooms sold on platforms, there are also a variety of ways through which hotels attempt to increase the benefits of platform bookings while minimizing the drawbacks of the platform logic. Hotels are not just passively submitted to platforms but attempt to optimize their use of them. Optimization strategies are about increasing revenue for hotels *on* platforms. We find that they do this in two ways. The first way seeks to regain some kind of control over the algorithmic sorting done by platforms. The second way seeks to optimize revenue from each transaction.

#### Playing the algorithmic sorting

Platforms control how they show hotels in their search results, using algorithms whose exact components are unknown to hoteliers. In order to optimize their platform use, hotels try to influence this sorting in spite of their limited knowledge of its exact functioning. A first way to do this is simply by becoming part of the platform’s “*preferred partner*” program. Many OTAs offer the possibility to benefit from a preferred partner status, associated with numerous market advantages. For instance, obtaining this status from Booking.com means paying 3% additional commissions on each reservation, that is, 15% instead of 12%. According to the explanations provided in the “Partner Hub” on Booking.com’s website, its preferred partner program offers an increased visibility to hotels in the ranking and allows them to display a “thumbs-up” icon that “acts as [a] seal of approval,” supposed to contribute to customer confidence. Hotels with such a status are promised to “getting up to 65% more page views and ultimately 40% more bookings”^4^. From the point of view of platforms, these programs appear to be a strategy to increase diversification and revenue through commissions. But from the point of view of hotels, the preferred partner status is a form of optimization. It is a way to buy some certainty with regard to one’s placing in the platform’s search results. Hotels pay to have some control over algorithmic sorting, and thus to be more visible.

Not every hotel can become a preferred partner, however: on the one hand, hotels need to be in an economic position where it is possible for them to pay higher commissions. On the other hand, it also depends on the platforms’ own policy. This status is generally only offered to a limited number of hotels in a given region and depends on selection criteria such as booking/cancelation rates or reviews. Furthermore, the privileged status program may also have an effect of increased competition among professionals in the tourism industry. This can be seen in the account given by the owner of a luxury hotel in a very touristic mountain resort:

We launched a campaign three years ago where we wanted all hotels [in the resort] to leave Booking.com’s preferred partners system. […] We called all the hoteliers who were preferred partners, and we almost managed to get all of them out, except that there were two who didn’t want to go out. And among the 40 [hotels that were members of the program], a dozen said “I only go out if everyone goes out”. But since there were two or three who refused to go out, well the 10 others said “Look, not everyone’s going out, so we’re not going out either.” […] We tried to get along with each other but it didn’t work.

In this example, the fear of some hoteliers to be disadvantaged compared to their local competitors resulted in the impossibility to implement a collective strategy consisting in reducing the commission rate—a typical prisoner’s dilemma.

Another important way how hotels try to gain some control over search results rankings are *review management* strategies. Here, hotels seek to obtain a better ranking and therefore a higher booking rate on OTAs by improving the hotel’s e-reputation through customer reviews. All our respondents acknowledge that customer reviews have taken on decisive significance and that they are a major selection criterion for guests. Most of them are very critical of this trend, refuting the ability of customers to make an objective judgment on the hotel offer and accusing OTAs of never verifying the veracity of the reviews—an observation that echoes previous studies (Cardon, 2015; Orlikowski and Scott, 2014). At the same time, some hoteliers see customer reviews as an opportunity to enhance their reputation and encourage their customers to evaluate them at the end of their stay. As the owner of a mountain family-run hotel expresses it, this effort makes it possible to distinguish oneself from the competitors but is highly work-intense:

You have to live with it, it’s part of the game. That’s why I always say that we have to drill the staff so that they hold out the little card “Please leave a comment”. And these are efforts that have been enormously made. It also helped us one thing, in 2013 we won the Welcome Award thanks to these reviews. So, yes, it’s very, very important.

The most common practice for getting positive reviews consists in offering an update to guests who have booked their room through Booking.com. A hotelier in the mountain region testifies:

On Booking.com, there are many more reviews because each customer automatically receives a request for evaluation, so the customer puts a comment. I know that there are hoteliers who systematically upgrade people who come by Booking.com, because of the reviews. […] I have a colleague who says “To Booking.com’s clients I always give a superior room”. Which isn’t normal, since this client already costs you more!

This example illustrates the difficult relationship between hoteliers and OTAs, where the aim is to minimize the cost of bookings made through OTAs while ensuring that the hotel has a sufficiently good e-reputation to appear well ranked on them. Other practices to improve ratings are more controversial, such as creating or buying fake customer reviews. As with the excerpt above, interviewees do not confess to such practices but frequently accuse others of using them.

#### Use of revenue management tools

Platforms attempt to control prices by forbidding hotels from offering lower prices on other (online) channels. Yet, the digital environment they provide also offers opportunities for hotels to improve their price management which would be difficult to implement otherwise. Inspired by the dynamic pricing systems developed in the aviation industry, revenue management (RM) or yield management practices are based upon a principle of differentiated pricing in real time. Revenue management simultaneously aims to optimize the occupancy rate and profitability (Ivanov, 2014). Platforms offer affordances to develop highly efficient RM practices, but to do so requires sophisticated technological skills and resources (Alrawadieh et al., 2021). Hotels use IT tools called channel managers (CM), intended to manage different online distribution channels, with direct interfaces to OTAs (see also below). In this way, the distribution, availability, prices of rooms, and other variables, such as booking conditions, can be largely automated. Such specialized computer software is developed by independent technical service providers who have come to play an increasingly important role in the hotel business. Once implemented, this technical integration saves precious time in terms of organizing the distribution of the offer.

Several CM software programs contain RM options, allowing direct application of dynamic pricing to online distribution on OTAs. Yet, the development of a RM strategy requires knowing enough of one’s customers to be able to divide them into segments and make real-time forecasts of the demand behavior of the various segments (Ivanov, 2014). The relatively high costs of using a CM and the required tech savviness for its use, can be an obstacle for small hotels. The manager of the mountain region’s luxury hotel comments on the difficult situation of small hotels, which do not have the same staff and organizational resources as his, to effectively manage their digital distribution:

In a small hotel […] which doesn’t have a marketing manager, a revenue manager or a sales manager […] it’s a single person who does the reception, the service, the check in, who answers the phone, who makes offers by email, etc. So, these hotels have more trouble doing their online sales properly. And they often become too dependent on one OTA.

Therefore, RM is mainly implemented by chain hotels and luxury hotels, which are often the only market actors to have the financial means allowing them to internalize the required skills or to outsource the management of this activity (Cetin et al., 2016b; Melis and Piga, 2017). Hotel chains seem to be clearly privileged in this area, as they have the possibility to centralize CM and RM resources and make them available to all member hotels (see also Richard, 2017: 59).

Another way for hotels to optimize revenue per transaction is simply by negotiating better (i.e., lower) commissions with platforms. This builds on a power relationship, and is mostly accessible to high-volume hotels, in general chains. In exchange for being on the platform and because they are guaranteed to bring in high volumes of transaction, chains are capable to negotiate better terms with platforms. In this specific case, we clearly observe a more equal relationship between the platform and hotels.

### Mitigation

The final category of strategies aims primarily to mitigate the effects of OTAs on hotels. Mitigation is not about optimizing the use of one OTA, nor about bypassing OTAs altogether; it is about taking advantage of the plurality of OTAs to vary the distribution of supply and the fixing of prices. If a hotel is only present on *one* platform and sells all its rooms through this channel, it becomes fully dependent on it, and a change in transaction conditions (such as a rise of commissions) can have dramatic consequences. From the point of view of the systemic analysis of “platform capitalism,” this is what platforms aim for—to become owners of digital marketplaces and profit from this position (Srnicek, 2017; Staab, 2019). But in reality, the situation on the market for hotel accommodation is (at least for now) quite different: it is characterized by a plurality of platforms as well as other, traditional distribution channels (hotels’ own websites, tour operators, etc.). This gives hotels the opportunity to switch distribution channels on or off, depending on the conditions they provide. One hotel manager told us, for instance, that he stopped working with Expedia because of their high commissions.

To some extent, hotels can thus do “platform shopping.” But unlike in cases of work platforms where gig workers can only be active on one platform at a time (Velthuis and Van Doorn, 2020) or are forbidden from working for several platforms in parallel, in the case of the hotel market a parallel presence on a plurality of channels is the norm. Just as in RM practices, IT tools are very important to make this strategy technically possible. The so-called CM allows hotels to stay up to date on all the bookings on the parallel channels they service, a task that is very complicated in an environment of fast and instant bookings. The CM automatizes the reservation process while allowing a control of the available rooms on each OTA.

Once a CM is in place, adding a new channel does not add any more costs and requires only a marginal amount of additional work, thanks to automatization. One no longer has to decide whether to market one’s hotel for one market segment or the other—for instance, to domestic, European, American, or Asian tourists. It is possible to combine all of this without it being necessary to switch off one channel in order to activate another one. Hotels can vary prices or other conditions such as minimum length of stay, depending on platforms/channels simply through the use of a single technical tool.

Being present on multiple platform channels thus allows hotels to mitigate their dependence on OTAs by taking advantage of their plurality and, related to this, the possibility this plurality offers to attract different segments of consumers. As with the RM techniques discussed in the previous section, this comes with a cost. In order to take advantage of it, hotels have to acquire necessary technological knowledge and practice. Larger hotels with specialized reservation officers have greater means to integrate this and actively use the software.

## Discussion and conclusion

The study identifies three different configurations of how market participants strategically respond to the platform logic. Table 1 gives an overview of these strategies and the mechanisms, resources (financial capacity or constraints; staff composition), and skills (technical, managerial, and relational) they rely on. While our small and snowball sample does not allow generalization about the quantitative distribution of the strategies identified, interviews give indications of which strategies hoteliers are more likely to implement according to the resources they have available. First, bypassing strategies channel market transactions away from the platform(s). These are therefore strategies that seek to reduce the hotel’s dependence on platforms by developing alternative means of capturing through which to attract bookings. Our empirical insights here suggest that qualification and valuation (Beckert and Aspers, 2011; Beckert and Musselin, 2013) are a key for how market participants can hope to limit their dependence on platforms. These forms of valuation rely on various forms of external resources and/or on “traditional players” who were partners before the arrival of platforms: local networks, third party certifiers (often professional or tourism associations) and hotel networks.

**Table 1.** Strategies and resources.

	Configuration	Strategies	Mechanisms	External resources needed	Cost	Technological skills	Characteristic for type of hotel
**Bypass**	*Outside* platform	*Value packages*	Create or reinforce alternative channels through qualification and valuation	In some cases, local networks	Low	Low	Small, family-run
		*Quality labels*		Third party certifier	Relatively low	Low	All hotel types
		*Loyalty programs*		Chain	Relatively low	High	Chains
**Optimize**	*On* platform	*Preferred partner programs*	Reduce uncertainty of dependence through control of algorithmic sorting	Platform	High	Low	All hotel types
		*Active review management*		None	Medium	Low	All hotel types
		*IT-supported revenue management*	Leverage technological affordances	Technical providers, software	High	High	Chains, luxury
		*Negotiation of commissions*	Power play	Chain	Low	None	Chains
**Mitigate**	*Between* platforms	*Presence on many channels through IT-supported channel management*	Reduce dependence by diversifying platform channels	Technical providers, software	High	Medium	All hotel types

Second, we find that organizations of this sector learn techniques and develop strategies to take advantage of the platform environment and optimize it in their favor. Such strategies thus play *on* the platforms themselves. On the one hand, hotels seek to gain some control over the algorithmic sorting the platforms make and thus to reduce some core uncertainties of the platform environment. They do so for instance by joining programs that platforms design to highlight certain hotels that have particularly good value, from the point of view of the platform (high conversion rates and low cancellation). It could be said that it is mostly in the interest of the platform and to some extent reinforces the platform logic (increasing dependence). However, from the point of view of hotels, it is a way of having some leeway over their positioning in the algorithmic sorting, and thus increasing their control over platform transactions, albeit by paying higher commissions. In addition, hotels do “review management” to obtain positive reviews. This strategy is accessible to all hotels and it is done in many, more or less legitimate forms. The impact of this form of optimizing is much less secure, however, since reviews are but one aspect of market hierarchies and algorithmic sorting.

Hotels also seek to leverage the technological affordances of platforms in their favor, by applying sophisticated tools of RM. This form of optimizing is rendered possible through third party technical providers developing software that takes advantage of the platforms’ technological environment and the possibilities this offers for data management and calculation. These technological skills are mostly available to large hotels and hotel chains with centralized RM services. Those same chains have yet another way to improve their profit through platforms: thanks to their high booking volumes, they are able to negotiate lower commissions and obtain better transaction conditions. The fact that they are capable of doing so shows that even in platformized markets, the direction of resource flows is not always clear cut; in this case, platforms depend on chain hotels at least as much as the latter depend on the former.

Third, we find that hotels are able to take advantage of the plurality of platforms. Hotels generally do not switch from one platform to the other, since it is possible for them to be present on a plurality of platforms at the same time. Specialized technology creates an interface with multiple platforms or distribution channels and thus allows organizations to take advantage of a situation of platform pluralism. Through this, dependence on one single platform is mitigated. This strategy comes with a certain entry cost, but is technologically not very sophisticated, and thus accessible to all types of hotels. However, it crucially depends on a situation of platform pluralism. As soon as one platform comes to dominate the market at large or a specific market segment, mitigation strategies lose their edge.

While the case study approach means that the empirical scope of the results is necessarily limited, the findings on strategic responses nonetheless yield important insights into the dynamics of contemporary platform economies. With this analysis, we contribute to the growing literature on platform economies, which has so far mostly analyzed the power of platforms to destabilize market orders and to impose a new digital logic on organizations (Kenney and Zysman, 2016; Kirchner and Beyer, 2016; Kirchner and Schüssler, 2019; Srnicek, 2017). Nuancing the picture of “algocracy” (Ajunwa and Greene, 2019) we expand studies on the capacities of workers to creatively adapt to and resist platform control (Vallas and Schor, 2020; Velthuis and Van Doorn, 2020) to a context where organizations are subject to processes of platformization in a market that already existed prior to the rise of digital platforms. We show that organizations (in our case, hotels) are not just passively submitted to the new logic platforms impose on markets but can develop ways to circumvent it and to use it in their favor.

In organizational sociology, scholars have long looked at the interplay between institutional pressures and organizations’ strategic capacities and reactivity (Espeland and Sauder, 2007; Oliver, 1991). Platforms create strong new forms of hierarchy and dependence on markets, but by identifying a range of strategic responses, we can see how and where the re-organization of markets by platforms is countered by market participants. On the one hand, the results of our study reveal contestation around the *reach* of platforms—the territory or market transactions that is actually organized by platforms. The collective response by the professional associations focused on limiting OTAs' capacity to extend their market organization beyond the actual platform environment, by prohibiting hotels from setting better prices on other channels. Meanwhile, bypassing strategies limit platforms’ reach by promoting transactions taking place outside of platforms. This allows hotels to access core resources—in this case, guests—through alternative channels, without passing through the platforms. Such bypassing of platforms seems to be a consistent feature of the platform economy, as it can be observed in other cases, too, such as when Airbnb hosts try to make direct arrangements with their guests or Uber drivers give their phone numbers to regular customers. But for markets that pre-exist the rise of platforms, bypassing is arguably facilitated because they can use previously existing channels. Our study also suggests that bypassing builds not only on price, but on different forms of valuation and qualification. Mitigation strategies similarly limit organizational dependence and the reach of singular platform, as hotels diversify their presence on platforms. Overall, strategies to limit the reach of platforms point at the plurality of market organizers that persists in platform economies and allows organizations to cope with this environment by reducing dependence.

On the other hand, we also find strategic responses that play out on the platforms themselves. Platforms’ technological affordances give hotels leverage to increase their revenue streams. Hotels also seek to gain some control of the algorithmic sorting in order to reduce uncertainties. While market participants cannot change the algorithmic rules, they find ways to influence their position, from joining programs to more crude forms of review management. Yet both of these optimizing strategies ultimately accommodate platforms more than countering them; they take place within rules that are set by the platforms without challenging nor changing them. The exception to this are the collective negotiations on commissions done by hotel chains; their high volumes can lead to a reversal of market hierarchies. A core element of the platforms business model—their skimming of a transaction fee—is changed in favor of market participants. Again, such insights are likely to be relevant to other cases of the platform economy, such as music streaming, where high-volume artists or labels may have a bargaining power that other actors lack; the latter are reduced to optimize their streams within the rules set by the platform (Prey, 2020).

The study also constitutes an important contribution to the understanding of the platform economy by differentiating between different types of market participants. Distinguishing small family-run middle-class hotels, independent luxury establishments, and chain hotels, we find that the effects that platforms have on them, as well as their capacities to strategically react to the rise of platforms, differ. Some studies on platform workers suggest that dependence on platforms is unequal and platforms create winners and losers, for instance, along technical skills, mobilization capacity or gender inequalities (Adams and Berg, 2017; Vallas and Schor, 2020; Wood et al., 2019). Similarly to this, we see that hotels are unequally equipped to counter platform power, although we also find that all hotels develop strategic responses. Some do so more quickly than others, some with conviction and others forced by the dynamics of seeking visibility. Overall, we observe differences that seem to reinforce longstanding inequalities and segmentations in the sector, between small and independent, often family-run hotels, and their larger competitors. This indicates that platformization tends to reinforce the market’s actors who already have an advantageous position and who possess a diversified set of resources. While platforms offer visibility to all hotels, the latter are much better equipped to develop strategies of optimization and mitigation, whereas bypassing strategies turn out to be somewhat more accessible to hotels with fewer resources.

This points at the importance of seeing the dynamics of platformization as a process. When platforms enter an existing market, they seem to offer better or equal opportunities to all hotels. But, as we have described, the benefits for some increase as they are better able to negotiate better rates, while for others the competition becomes fiercer. At this stage, however, we cannot know whether this competition may lead to the disappearance of establishments, or whether it affects establishments that were already fragile before the appearance of OTAs. In addition, the analysis of this competition struggle would need to include also the impact of non-professional competitors entering the market through peer-to-peer platforms like Airbnb. All these aspects of platformization, which can be observed in many different markets, certainly deserve further investigation: To which extent does platformization not only create a monopolistic power on platforms’ side, but also increase the concentration of organizations, by favoring incumbent organizations? Since platforms tend to stress the idea that all market players benefit from their efficient match-making, it is especially important that this process of market concentration be further investigated.

The processual nature of platformization also becomes apparent in the interplay between platforms and the strategic responses of organizations. For instance, our study shows how hotels develop bypassing strategies building on pre-existing networks, for instance, using their connections in local networks to develop packages that include ski passes in winter sports resorts. But the competition to benefit from network effects is currently increasing. While OTAs started out exclusively as a way to match supply and demand for rooms, they are now also including new options by aggregating complementary booking tools for visits, activities, passes offered sometimes by the same stakeholders the hotels rely on. The capacity of OTAs to increase connections with a lot of other “complementors” is indeed an indication of their openness, their boundary resources (De Reuver et al., 2018), and their growing market position (Lynskey, 2017). And through this characteristic feature of platforms, bypassing strategies relying on alternative forms of qualification and valuation may quickly become less efficient. Finally, the platform power will also result from the regulatory framework that will affect the transactions between platforms and hoteliers. New pieces of regulation may change the balance of power in the future. In the hotel sector, regulation today mainly concerns legal rules on room-price setting on and outside of platforms, but it can expand to legal control of commissions and contracts or to tax policies towards platforms.

## Notes

1 For hotel owners and managers, Airbnb adds new competitors, mainly non-professionals offering private accommodations that were not marketized beforehand. A very small minority of hotels seek to gain visibility by proposing rooms on this type of platform, hoping to attract customers attached to sharing economy. In addition, the main regulatory issues concern the extension of professional norms (hygiene, safety, and tourist tax) to the new non-professional competitors (Guttentag, 2015).

2 According to one study (Schegg, 2019) the average commission for Booking.com in Switzerland is 13%.

3 In many other European countries, equivalent institutions were called upon by trade associations of the hotel sector to rule on these clauses.

4 https://partner.booking.com/en-us/help/growing-your-business/increase-revenue/all-you-need-know-about-preferred-partner-program (accessed 28 October 2020)
